# Human Norovirus Proteins: Implications in the Replicative Cycle, Pathogenesis, and the Host Immune Response

**DOI:** 10.3389/fimmu.2020.00961

**Published:** 2020-06-16

**Authors:** Claudia P. Campillay-Véliz, Jonatan J. Carvajal, Andrea M. Avellaneda, Darling Escobar, Camila Covián, Alexis M. Kalergis, Margarita K. Lay

**Affiliations:** ^1^Departamento de Biotecnología, Facultad de Ciencias del Mar y Recursos Biológicos, Universidad de Antofagasta, Antofagasta, Chile; ^2^Departamento de Genética Molecular y Microbiología, Facultad de Ciencias Biológicas, Millennium Institute on Immunology and Immunotherapy, Pontificia Universidad de Chile, Santiago, Chile; ^3^Departamento de Endocrinología, Facultad de Medicina, Pontificia Universidad Católica de Chile, Santiago, Chile

**Keywords:** human norovirus, proteins, replication, pathogenesis, immune response

## Abstract

Human noroviruses (HuNoVs) are the cause of more than 95% of epidemic non-bacterial gastroenteritis worldwide, with some lethal cases. These viral agents affect people of all ages. However, young children and older adults are the highest-risk groups, being affected with the greatest rate of hospitalizations and morbidity cases. HuNoV structural proteins, especially VP1, have been studied extensively. In contrast, the functions of the non-structural proteins of the virus have been undescribed in depth. Studies on HuNoV non-structural proteins have mostly been made by expressing them individually in *in vitro* cultures, providing insights of their functions and the role that they play in HuNoV replication and pathogenesis. This review examines exhaustively the functions of both HuNoV structural and non-structural proteins and their possible role within the viral replicative cycle and the pathogenesis of the virus. It also highlights recent findings regarding the host's innate and adaptive immune responses against HuNoV, which are of great relevance for diagnostics and vaccine development so as to prevent infections caused by these fastidious viruses.

## Introduction

Human noroviruses (HuNoV) are the main cause of non-bacterial gastroenteritis worldwide, producing high morbidity and mortality rates (1, 2). It has been estimated that HuNoV causes 699 million illnesses and 219,000 deaths per year worldwide (3). Even in a developed country, such as the United States, it is estimated that the burden of the disease caused by HuNoV infection produces 570–800 deaths, 56,000–71,000 hospitalizations, 400,000 visits to the emergency room, 1.7–1.9 million ambulatory visits to health centers, and 19–21 million total diseases per year (4). Although HuNoV can infect people of all ages (5–7), among the high-risk populations are children under 5 years of age—who have the highest rates of medical care associated to HuNoV (4), the elderly—who have the highest mortality rates (4), immunocompromised patients (8, 9), and travelers visiting developing countries (10, 11). HuNoV produces rapid and self-limited acute gastroenteritis characterized by the development of symptoms such as vomiting, watery diarrhea with or without nausea, and abdominal cramps (12).

HuNoV belongs to the family *Caliciviridae*, specifically to the genus *Norovirus* (2), which has recently been subdivided into 10 different genogroups (GI–GX) (13). Only GI, GII, and GIV infect and cause acute gastroenteritis in humans. These genogroups are classified according to the similarity of the highly conserved regions of the RNA-dependent RNA polymerase (RdRp-NS7) or the conserved amino acidic regions of the capsid protein, VP1 (14). The HuNoV genogroups are subdivided into different genotypes based on mutations that are produced by selective pressure and/or other factors to which these viruses are exposed (14, 15). These mutations produce different variants or strains of the same genotype. For example, within the GII.4 genotype, Sydney and New Orleans are the most prevalent variants worldwide (16). These variants may also recombine with others, giving rise to new recombinant strains, including the pandemic GII.P16–GII.4 Sydney strain, which is highly contagious (17). Although the most prevalent genotype worldwide is GII.4 (2, 15), cases caused by other highly infectious genotypes, such as GII.17, have been detected (17, 18).

There are no commercial vaccines or specific antivirals available to prevent HuNoV infections. The fact that there is no availability of small animal models that emulate the human disease so as to perform pre-clinical studies makes even more difficult the development of vaccines or preventive therapies against these viruses. Recently, there had been *in vitro* culture models developed for the replication of different HuNoV strains, specifically GII.4-Sydney HuNoV replicates in human B cells (19). Moreover, GII.3 and different GII.4 variants replicate in human intestinal enteroid monolayer cultures (20). Most recently, an *in vivo* replication model for GI and GII HuNoV genogroups in zebrafish larvae has also been developed (21). Nevertheless, limited information regarding the viral replication cycle and the immune response against HuNoV is available. Knowledge in these areas will allow a better understanding of viral pathogenesis and the design of efficient strategies to develop a safe vaccine against these fastidious enteric viruses (22, 23). In this review, we examine recent information on the functions of the HuNoV structural and non-structural proteins and their role in the viral replicative cycle and pathogenesis, as well as insights of the host immune response against these viruses.

## Characteristics of Human Noroviruses

### Structure and Genome of HuNoV

**Figure 1:**
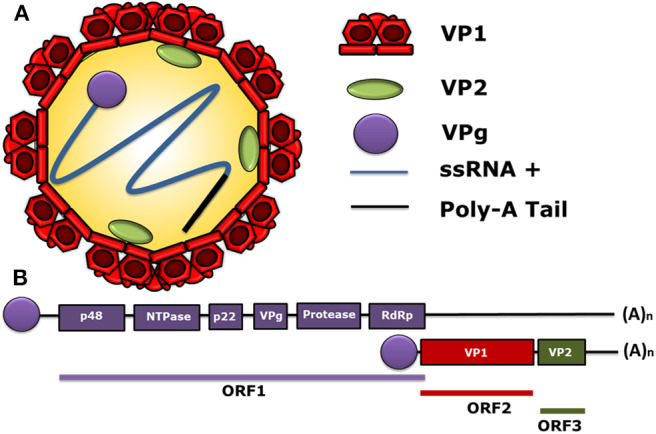
Molecular structure of human norovirus (HuNoV). **(A)** A schematic representation of the human norovirus virion showing its 90-dimer protein surface of the VP1 structural protein of the capsid. The structural protein VP2 (1–8 proteins per virion) is shown within the viral capsid. The non-structural VPg protein, covalently bound to the 5′ end of the RNA genome (ssRNA+) in a positive sense and a poly-adenine tail at the 3′ ends are also displayed. **(B)** General scheme of the HuNoV genome organization. ORF1 encodes the non-structural proteins: p48, NTPase, p22, VPg, Protease, and RdRp. The ORF2 encodes for the major structural protein VP1 and the ORF3 encodes for the minor structural protein VP2. The subgenomic RNA bound to VPg encoding VP1 and VP2 is indicated below the ORFs. VPg is represented as a circle linked to genomic and subgenomic RNAs.

The first sequenced genome of HuNoV was the one of the Norwalk Virus (NV), belonging to genogroup I (GI), in the early 1990's. The HuNoV genome is a positive-sense single-stranded RNA (ssRNA+), with 7.5–7.7 kb covalently bound to a non-structural (NS) protein, called viral protein genome-linked (VPg), at the 5′ end and polyadenylated at the 3′ end (24). The HuNoV genome contains three open reading frames (ORF). ORF1 encodes a polyprotein with a molecular weight of 200 kDa (25), which is composed of six NS proteins. Starting from the 5′ end, these NS proteins are p48, nucleoside-triphosphatase (NTPase), p22, VPg, protease, and the RNA-dependent RNA polymerase (RdRp) (26–28). Likewise, ORF2 encodes the major structural protein of the viral capsid VP1, and ORF3 encodes the minor structural protein of the capsid VP2 (26–28). The structural and the genomic characteristics of HuNoV are displayed in Figure 1, and the nomenclature of its viral proteins is shown in Table 1.

**Table 1:** Table of the known functions of the structural and non-structural proteins of HuNoV.

**Proteins**	**Nomenclature**	**Function**	**References**
NS ½	• p48 • N-terminal	° Modulate the activity of the viral polymerase (RdRp). ° Alter multiple immune systems. ° Function as an agonist of cellular secretory pathways.	(29–31)
NS 3	• NTPasa • 2C-like^*^ • p41 (p40) • Nucleoside triphosphatase like 2C	° Similar activity to helicase ° Union and hydrolysis of nucleoside triphosphates. ° It helps in the synthesis of RNA *in vitro* carried out by the RdRp.	(32–34)
NS 4	• p22 (p20) • 3A-like^*^	° Mimics an export signal for vesicles coated with COPII from the RER. ° Prevents the union of vesicles with the Golgi Apparatus. ° Ability to induce disassembly of the Golgi Apparatus. ° Antagonistic activity of the secretion of proteins that depend on the Golgi Apparatus.	(33, 35, 36)
NS 5	• VPg • Virus protein, linked to the genome	° Interact with initiation factors such as eIF3. ° Direct the synthesis of viral proteins.	(33)
NS 6	• Protease • Protect like 3C • Pro • 3C-like^*^ • 3CLpro • ProPol^*^ • NS6^pro^	° Responsible for processing the viral polyprotein.	(33)
NS7	• Polymerase • Pol • 3Dpol^*^ • RdRp • ProPol^**^ • NS7^pol^	° Translation of viral RNA.	(33, 37)
VP 1	• Viral protein 1 • The major protein of the capsid viral	° Structural protein. ° Binding of the virus to HBGA. ° Immune escape.	(22, 38)
VP 2	• Viral protein 2 • The minor protein of the capsid viral	° Translation of viral RNA. ° Structural protein. ° Stability of the viral capsid.	(28)

* *These nomenclatures are made based on the similarity they have with the proteins of norovirus with those of Picornavirus*.

** *This term refers to a protease precursor protein and polymerase. The polymerase in this state is an immature polymerase but it has all the functions of the already excised mature polymerase (ProPol, NS6-7)*.

### Characteristics and Functions of the Structural Proteins of HuNoV

#### VP1

VP1 is the main structural protein of the capsid of HuNoVs. It has an approximate molecular weight of 58 kDa, determined by expressing the NV ORF2 in a baculovirus expression system (39). Moreover, the three-dimensional structure of the NV capsid revealed that the empty capsid has a diameter of 38 nm, with an icosahedral symmetry of *T* = 3, which is composed of 90 VP1 dimers (40). Likewise, a structural analysis of the NV capsid using X-ray crystallography showed that it has two distinct substructures, the shell (*S*) and the protruding (*P*) domains, which are linked by a flexible hinge (41).

The *S* domain of NV VP1, which comprises from the N-terminal end to residue number 225 of the protein, conforms with the icosahedral shell of the virus and possesses a Pro-Pro-Gly sequence, which is conserved among different HuNoV viruses (41). This domain has been associated with the development of a smooth layer of virus-like particles (VLPs). NV VLPs lacking the expression of the *P* domain have a diameter slightly smaller than 30 nm, which is the expected size for the native NV structure, suggesting that the *S* domain has the necessary components to start the HuNoV capsid assembly (42). Also, since the *S* is a conserved domain among genotypes of a same genogroup, it is used for the identification of norovirus genogroups (41).

On the other hand, the *P* domain of NV VP1, which comprises from residue 225 to the C-terminal end of the protein, is the domain that protrudes from the shell of the capsid (41). The *P* domain contributes to the control of the size and the stability of the HuNoV capsid because it establishes intermolecular contacts between dimeric VP1 subunits (42). It has also been observed that the *P* domains do not form VLPs. Nevertheless, the *P* domain can bind to the histo-blood group antigens (HBGAs) using the same patterns as the native viral capsid, playing a role in the virus–cell receptor interaction (43). This domain is further divided into two subdomains: P1 and P2 (41).

The subdomain P1 is constituted by residues 226–278 and 406–520 of the *P* domain (41). In contrast, the P2 subdomain is constituted by residues 278–406 of the *P* domain, with a length of 127 amino acids (aa), which is inserted in the P1 domain (41). The most variable region of the *P* domain is the subdomain P2, which is extended from the *S* domain to the outside of the capsid and is located in the middle part of the structural protein VP1 (28, 44). In a study of HuNoV VP1, the sequence diversity in the P2 region was determined by sequence analysis techniques and homology models using the amino acid sequences of the GII.4 VP1 protein. The results of this study indicated that two sites within the P2 region appear to be involved in the generation of variants associated with epidemics due to the presence of amino acid substitutions. Moreover, these sites are exposed on the surface of the viral capsid, suggesting that the P2 region could exhibit specific epitopes of each GII.4 variant (44). VP1 is the target protein for antibodies directed to the *S* and the *P* domains (41), the P2 subdomain being the region with the highest exposure of the viral particle, which allows its interaction with the HBGAs and with possible neutralizers (45, 46).

Interestingly, NV VP1 can self-assemble, forming VLPs of similar size and appearance with the native structure of the virus capsid by expressing it in insect cells, using the baculovirus system (39). Furthermore, in VLP binding and internalization assays in human and animal cell lines, it was observed that NV VLPs bind to cell membranes. However, only 1.4–6.8% of the VLPs were internalized in the tested cells. Besides that, the monoclonal antibody NV8812 exhibited the ability to block the binding of NV VLPs to those cells. This antibody specifically binds to the C-terminal region at residues 300–384 of NV VP1, suggesting that this region could be involved in VLP–cell binding (47).

The amino acid sequence of the VP1 protein is used to genetically group HuNoVs as follows: those with differences <14.3% are classified in the same strain, those with differences between 14.3 and 43.8% are classified in the same genotype, and those with differences between 45 and 61.4% are classified in the same genogroup (48). The GII.4 genotype is the most prevalent HuNoV in the world and the one with the greatest variability, with emerging new variants of it every 2–5 years, due to changes in its antigenicity and alterations in ligand binding patterns (49). Indeed the escape of herd immunity is promoted when these changes in viral antigenicity are generated, resulting in new HuNoV outbreaks in the population (50). Various mechanisms to explain the variability of HuNoVs have been proposed, among which stands out the changes in antigenicity, specifically in the subdomain P2 of the VP1, due to the strong selective pressure that this subdomain undergoes when presenting antigenic epitopes and receptor-binding regions of the host (14, 46, 50, 51), and the homologous recombination in the ORF1/ORF2 overlap region, generating intergenotype, and intragenotype recombinants (46, 52, 53).

Taken together, the VP1 is a complex structural protein of the capsid that also appears to play a fundamental role in the interaction of HuNoVs with the host cell and is essential for the classification of these viruses.

#### VP2

The viral protein 2 (VP2) is the minor structural protein of HuNoV, with a molecular weight of 29 kDa and which is located inside the viral capsid. VP2 is one of the less-studied HuNoV proteins. Nevertheless, it was recently determined that VP2 interacts with VP1 at the amino acid 52 of its *S* domain, which corresponds to an isoleucine located within the sequence motif IDPWI that is highly conserved across norovirus genogroups (28).

A comparative analysis of the genomic regions of HuNoV sequences determined that the evolutionary rate of the region encoding the VP2 protein (GII.P4 Den Haag 2006b) is of 8.99 × 10^−3^ substitutions/site/year, being higher compared to the evolutionary rate of VP1 (5.94 × 10^−3^ substitutions/site/year), suggesting that this protein is under positive selection and likely involved in interactions with host restriction factors (54).

Some studies have reported that VP2 is not essential for the formation of VLPs (42). On the contrary, other studies have suggested that this protein stabilizes and coordinates the formation of VLPs (28, 55). Indeed the absence of VP2 decreases the stability and the homogeneity of the size of NV VLP (28). It has been likewise suggested that the internal location of VP2 in the NV particle and its highly basic nature are consistent with the potential role of VP2 in the capsid assembly and the genomic encapsidation of NV (28). Furthermore, *in vitro* studies have shown that the co-expression of VP1 and VP2 increased the expression of these proteins compared to when they are expressed individually. This effect occurs independently of the presence of NS proteins (56). Remarkably, in the same study it was reported that the co-transfection of VP1 and VP2 of GII.4, testing different variants, was found in the cell nucleus of human 293T cells. Moreover, it was shown that VP1 was expressed, located throughout the cytosol, in the absence of VP2. In contrast, VP2 was expressed in the absence of VP1 and was observed to be located in the nucleus (56). In support of this notion, a predicted specific nuclear localization signal has been reported in the HuNoV ORF3, suggesting that VP2 could also serve as a VP1 helper protein, directing this protein complex to the nucleus. This complex could play a role in interfering with the normal cellular processes in the cell nuclei, such as transcriptional and chromatin remodeling processes. Further research is still required to confirm these possibilities because these HuNoV proteins were not found in the nucleus of insect cells (26, 56).

Another *in vitro* study showed that VP2 negatively regulates the activity of the HuNoV RdRp (57), suggesting a role in HuNoV replication for this structural protein. Interestingly, this RdRp regulation activity is a characteristic shared by VP1, P48, and VP2 proteins (57). Finally, HuNoV VP2 can also have a role in modulating the immune response of the host based on studies done with murine norovirus (MNV) VP2 protein. However, this possibility has not yet been proven for HuNoV (35). Therefore, VP2 appears to be a versatile structural protein that may have many different functions in the replicative cycle and the pathogenesis of HuNoV.

### Characteristics and Functions of Non-structural Proteins of HuNoV

#### p48 or N-Terminal Protein

The p48 protein, also denoted as N-terminal (NS1-2), is a characteristic protein of the *Norovirus* genus, located at the N-terminus of the polyprotein. When it is cleaved by the viral protease, it results in a mature NS1-2 protein. In GI.1, this protein has 398 aa (58, 59) and a molecular weight of 37 kDa (60). The length of GII p48 is the same as the one of GI.1, but its weight varies between 45 and 48 kDa (61). The evolutionary rate of the region that encodes for the p48 proteins of the GII.P4 Den Haag 2006b strain is 6.60 × 10^−3^ substitutions/site/year, which is higher than the evolutionary rate determined for its entire genome (6.15 × 10^−3^ substitutions/site/year) (54). In an alignment of the p48 protein sequences from different norovirus genogroups, it was determined that the HuNoV Sydney p48 shares 42% identity with NV p48 (GI), 36% with Jena p48 (GIII), and 37% with MNV (GV) (29). Even though these proteins share a low similarity, through an *in situ* analysis it was determined that the HuNoV p48 protein has amino acid sequence characteristics like the MNV p48 protein that include: (1) a disordered region of proline-rich N-terminal, containing the alleged immunogenic regions for MHC-I binding, (2) a transmembrane hydrophobic domain at the C-terminal end, (3) H-box and NC sequence motifs of the permutated NlpC/P60 family of circular peptidases that adapt different enzyme capacities within the same structure, improving the stability and the reducing degradation caused by proteases, and (4) caspase cleavage and phosphorylation sites, which in eukaryotic cells are involved in the regulation of the cell cycle, apoptosis, and activation of the immune system (29). The specific function of the p48 protein of the different HuNoVs within the viral replicative cycle and its role in HuNoV pathogenesis in the host have not yet been elucidated. Moreover, a confocal microscopy analysis in HeLa and Crandell–Rees feline kidney cells transfected with a vector plasmid that expresses the NV p48 protein revealed that this protein can co-localize with the trans-Golgi network protein markers and it induces the disassembly of this cellular organelle in discrete aggregates (58). Besides that, the expression of p48 as a fusion protein in COS-7 cells, an African green monkey kidney fibroblast-like cell line, revealed that this protein binds to the protein A associated with the vesicle-associated membrane proteins (VAP-A) located at the membranes of the endoplasmic reticulum (ER) and the trans-Golgi network. Also, VAP-A can bind to the SNARE protein complex that regulates vesicular transport by the interaction of p48 with VAP-A. The p48 protein is localized in the vesicle membranes of the ER, the trans-Golgi network, and the endosomes, suggesting that the interaction of p48 with VAP-A could act as a scaffold for the assembly of replication complexes (30, 58). Hence, a transcriptomic analysis determined, by expressing the HuNoV p48 protein in murine monocytes, revealed that it can interfere in many intracellular pathways, such as those involving the Jak-STAT, MAPK, p53, and PI3K-Akt signaling pathways. Additionally, HuNoV p48 has been shown to interfere with apoptosis, Toll-like receptors (TLR) signaling pathways, and the production of chemokines and cytokines (29). Thus, the p48 protein can play an important role in the HuNoV replicative cycle by participating in the development of the viral replication complex and by hampering the normal function of cellular signaling pathways, as well as, the activation of the immune response induced by the viral infection.

#### NTPase

The NTPase protein, also denoted as NS3, is cleaved by the viral protease at the 331 and 696 aa of the polyprotein, resulting in a mature protein with a length of 366 aa and a molecular weight of 40 kDa (61). It has been estimated that the evolutionary rate of the NS3 protein from a collection of HuNoV GII.4 genomic sequences is ~5.41 × 10^−3^ substitutions/site/year (54). The NTPase activity of this protein was described in a study where the protein was expressed and purified from the Southampton virus (SHV) belonging to genogroup 1 (GI) of the *Norovirus* genus, determining that this protein can bind to nucleoside triphosphates (NTP) and hydrolyze them (32). Also, a comparative analysis of the sequence of the NTPase protein of SHV with the NV NTPase protein and the Enterovirus 2C protein determined regions with high homology, suggesting a functional relationship between the HuNoV NTPase protein and the 2C protein of Enterovirus (32). Furthermore, NV NTPase possesses fundamental enzymatic activities that play a significant role in viral replication, such as (a) NTP-dependent helicase activity for unrolling of RNA helices, (b) NTP-independent chaperone activity for remodeling of the RNA structure and facilitating the annealing of the RNA chains, and (c) collaboration in RNA synthesis carried out by NS7 (62). Significantly, the subcellular localization of the GII.4 NTPase protein was determined through a confocal immunofluorescence analysis in human melanoma A7 cells, determining that this protein can form vesicular and non-vesicular structures in the cell cytoplasm (63). Besides that, the non-vesicular form of NTPase can be located in the ER or the membrane of the cellular mitochondria. The amino acid 179 of the N-terminal region is necessary for the formation and the localization of vesicles in the ER (63). On the other hand, for mitochondrial localization, the NTPase C-terminal region of GII plays a significant role, even when the N-terminal NTPase region is completely removed (63). However, this activity is not observed in the GI viruses (NV); thus, the NTPase mitochondrial location differs between HuNoV genogroups (63). Furthermore, it has been reported that HuNoV GII.4 NTPase locates in different cell membrane compartments in the cellular secretory pathway and that it is associated with the storage of intracellular lipids (64). Notably, the NTPase function can be improved by other NS proteins such as p48 and p22. Indeed the co-expression of these proteins with NTPase enhances the apoptotic activity induced by the HuNoV NTPase (63). Thus, the HuNoV NTPase protein could play a significant role in the replication and the pathogenesis of the virus.

#### P22

p22, also called p20 (NS4), when excised from the polyprotein by the viral protease, results in a mature NS protein of 20–22 kDa, depending on the genogroup (36, 61). The evolutionary rate of the genomic region that encodes the p22 protein of the strain GII.P4 Den Haag 2006b of HuNoV is ~8.21 × 10^−3^ substitutions/site/year, which is higher than the evolutionary rate determined for the complete genome, being one of the most variable genomic regions of this strain (54).

Although the functions of p22 are not completely known, some of its characteristics have been elucidated. Indeed it has been shown that NV p22 harbors a YXΦESDG motif (36), which mimics an export signal for vesicles coated with COPII that migrate from the rough ER and prevents these coated vesicles from fusing with the trans-Golgi network (36). In this way, p22 interferes with the protein secretion and post-translational modification pathways. It should be noted that this motif is highly conserved among different genotypes of the GI and the GII genogroups (36).

The different rearrangements of vesicular and tubular membranes play a critical role in the development of viral replicative complexes in RNA(+) viruses. Indeed in an *in vitro* study using the Huh7 cell line, it was shown that the GII p22, when expressed individually, can constitute single-membrane and double-membrane vesicles, contributing to membrane alterations within the cell to promote, together with the p48 and NTPase, the formation of the viral replication complex (64).

Remarkably, HuNoV p22 is very similar to picornavirus 3A and MNV p22 proteins, sharing with them some characteristics (65). HuNoV p22, like its picornavirus 3A and MNV counterparts, participates in the induction of trans-Golgi network dismantling, although the exact mechanism of action remains to be elucidated (36). Also, it has been reported that p22 has the following activities: (1) it acts as an antagonist in Golgi-dependent protein secretion (36); and (2) it acts as an antagonist of the immune response by altering the interferon and other cytokine signaling pathways after viral infection (35, 36). Thus, the HuNoV p22 protein could play a crucial role in the pathogenesis and the replication of these viruses by disrupting the normal functions of the host cell and the activation of the immune response. Besides that, it promotes the replicative viral complex assembly.

#### VPg

The HuNoV VPg (NS5) genome-linked protein is the NS protein that is covalently linked to the 5′ end of the viral RNA genome. VPg is cleaved by the HuNoV protease at residues 876 and 1,008 of the polyprotein, resulting in a mature 132-aa protein with a molecular weight of 15.8 kDa (MD145-12 GII. 4) (61). The evolutionary rate of the genomic region that encodes the VPg protein of the strain GII.P4 Den Haag 2006b is ~5.95 × 10^−3^ substitutions/site/year (54).

It has been described that the HuNoV GII.4 ProPol form can nucleotidylate the VPg protein at tyrosine residue number 27 in the presence of Mn^+2^, resulting in the binding of VPg to the viral RNA, thus facilitating the HuNoV genome replication by acting as a primer in viral RNA synthesis, which is mediated by the RdRp (66).

Moreover, unlike the eukaryotic genome, the NV mRNA lacks a coding region for a cap structure and it does not have an internal ribosome binding site. Therefore, it was described that VPg can participate in the initiation of viral RNA translation by binding to the cell translation initiation factor eIF3 (67) and interacting with the cap-binding complex eIF4F (68).

In cell lines, it was described that the interaction between NV VPg and the “core stress granule component” (G3BP1) is important for viral protein synthesis and replication since this complex binds to ribosome 40S, providing a competitive advantage over the host cell RNAs (68).

Furthermore, in an *in vitro* transfection study in RAW-Blue murine macrophages, it was determined that VPg of different HuNoVs genogroups can induce cell cycle arrest in phases G0/G1. The VPg of GI and GII increases the percentage of G0/G1 cellular phases to 85 and 60%, respectively. Furthermore, it was identified that the cell cycle arrest is induced by the conserved motif KGKxKxGRG in the N-terminal region which is found in the VPg proteins of all the norovirus genogroups, except for GIII. These findings demonstrate that the N-terminal end of VPg is essential to arrest the cell cycle in G1/G0 and promotes HuNoV replication (69). Therefore, the interactions of VPg with the RdRp and cellular components of the host play an important role in the viral replicative cycle and the pathogenesis of HuNoV.

#### Protease

Protease plays a fundamental role in viral activities by cleaving the proteins involved in genome replication, virus pathogenesis, and the interaction with host cellular factors. The HuNoV protease, also called 3CL Pro (NS6), is similar to the 3C protease of *Picornavirus* (59), which processes the NS proteins by cleaving them from the viral polyprotein (61). It has an approximate molecular weight of 19.4 kDa (70), and it has been estimated that its evolutionary rate from a collection of GII.4 sequences is ~6.03 × 10^−3^ substitutions/site/year (54). The cleavage process of the polyprotein by the HuNoV protease consists of “early” cleavage sites (p48/NTPase and NTPase/p22), which are more efficiently cleaved than the “late” cleavage sites (p22/VPg, VPg/Pro, and Pro/Pol) (59, 71). Additionally, the HuNoV protease has been characterized as a chymotrypsin-like protease (61), containing in its active site Cys139, His30, and Glu54 catalytic residues (72, 73). Moreover, the optimum pH and temperature for its proteolytic activity are around 8.6 and 37°C, respectively (74).

An analysis of the GII.4 protease crystal structure shows that this protein presents changes in the S2 substrate-binding pocket and the active site (catalytic triad residues) compared to those sites in the GI homologous protein. Another characteristic of the active site of the GII.4 protease is that there is a conserved arginine that interacts with the catalytic histidine, which restricts the access of inhibitors or substrates to the S2 pocket and makes the proteolytic activity of this protease sensitive to pH changes (75). Thus, the GI protease inhibitors may not be effective for the GII.4 protease.

Additionally, the HuNoV protease has an intracellular precursor form, called ProPol, which has been shown to have protease and polymerase activities, partially processing the polyprotein encoded by ORF1 (37, 76). Interestingly, ProPol has an optimal activity at pH between 8.6 and 9.0, increasing its activity at a higher pH than the viral protease by itself. Moreover, ProPol has higher activity (64%) than the mature protease (34%) when the pH decreases to around 6.8. Also, both enzymes are inhibited when exposed to NaCl, KCl, MgCl, and MnCl_2_. Nevertheless, the HuNoV ProPol is more sensitive to CaCl_2_ exposure than the protease itself (77). Besides that, the GII.4 ProPol can nucleotidylate the VPg protein in the presence of Mn^+2^ (66).

Furthermore, when comparing the protease activity to that of ProPol using peptides representing the five cleavage sites of the HuNoV polyprotein, it was shown that ProPol has a protein cleavage efficiency equal to or greater than the viral protease itself at all the polyprotein cleavage sites (77). Therefore, the ProPol precursor can function as the dominant form of the HuNoV protease.

#### Polymerase

The HuNoV polymerase protein, also called Pol (NS7), is a RdRp whose main function is the replication of the viral genome. This protein is cleaved by the HuNoV protease at residues 1,190 and 1,699 of the polyprotein, resulting in a mature protein of 509 aa and 56.8 kDa in molecular weight (MD145-12 GII.4) (61). It has been described that the GII.4 MD145-12 ProPol precursor has the functional activities of polymerase and proteinase, which are conserved within some members of the *Caliciviridae* family, suggesting that ProPol is a bifunctional complex that acts during viral replication (37, 61). The three-dimensional structure of the NV Pol protein has been described with resolutions of 2.17 and 2.95 Å in the absence and the presence of metals, respectively. Also, it has been described that the structure of the NV Pol protein is very similar to that of other viruses, varying only in the sense that this viral Pol has a carboxyl terminal in the slit of the active site that could be involved in the synthesis of viral RNA (78).

Phylogenetically, the Pol region has been used to designate the P types of HuNoVs, which have been determined to be composed of 60 P types (14 GI, 37 GII, two GIII, one GIV, two GV, two GVI, one GVII, and one GX), two tentative P groups, and 14 tentative P types (13).

Furthermore, an analysis of the molecular evolution of the region coding for the Pol protein, based on different HuNoV GII genome sequences, suggests that the common ancestor of the Pol region of GI, GII, GIII, and GIV diverged ~1,150 years ago. Likewise, the Pol region of GII diverged from GIV about 570 years ago, giving origin to three main lineages of HuNoV GII (79). Besides that, it has been estimated that the evolution of the HuNoV GII.4 Pol protein presents ~4.33 × 10^−3^ to 8.98 × 10^−3^ substitutions/site/year (54). It was identified that the GII Pol protein has several amino acid substitutions (39–107 sites) in different genotypes, while the negative selection sites were found to be different for each genotype (79). These results suggest that the Pol region of HuNoV GII has a rapid evolution with several amino acid substitutions and that the evolutionary mechanism may be different for each genotype (79). Therefore, the HuNoV polymerase is not only a pivotal protein for virus replication and transcription processes but also a useful NS protein for the classification of the *Norovirus* genus.

## Host Susceptibility Factors to HuNoV

The recognition and binding of HuNoVs to HBGAs has been described as the primordial step that precedes the entry of a viral particle into the cell (80). HBGAs are polymorphic glucans found on the cell surface of red blood cells or the epithelial cells of some tissues (81). In HuNoV, the HBGA binding sites are specifically located in the P2 subdomain of the P domain of VP1, which is highly variable (43, 56). The variability of this binding site is found across the different norovirus genogroups. This is due to the fact that each HuNoV genogroup/genotype binds to different HBGAs (14, 31, 82). Additionally, an *in vitro* study has shown that the interaction of HuNoV with HBGA is of low affinity and does not induce a significant structural rearrangement in VP1 (83).

There are susceptibility studies that indicate that several individuals are resistant to HuNoV infection (84). The HBGA generation is mediated by the enzyme fucosyltransferase 2 (FUT2), which is encoded by the *fut2* gene. Individuals who present this functional gene are called secretors and are susceptible to HuNoV infection. In contrast, those who do not have this functional gene are called non-secretors and are resistant to HuNoV infection (81). HBGA, as a pivotal factor in the susceptibility of individuals to virus infection, has also been reported in other enteric viruses such as rotavirus. Indeed it has been shown that secretor-positive (FUT2+) individuals are 26.6 times more likely to be infected by rotavirus than non-secretor individuals (52, 85, 86).

The first link between HBGA variability and HuNoV infection was suggested from clinical studies of volunteers infected with NV (87), in which it was shown that the virus recognizes a determined group of HBGAs (31). There are at least four specific binding patterns of HuNoV strains based on the ABO, secretor, and Lewis blood types of saliva donors (88). Moreover, the HuNoV-specific binding patterns to HBGAs can be classified into two important groups according to their interactions with human HBGA (A/B, H, and Lewis) (31): (i) HuNoVs that have HBGA type A/B binding patterns recognize the A and/or B and H antigens, but not the Lewis antigens; and (ii) HuNoVs that have Lewis binding patterns bind only to Lewis antigens and/or the H antigen (31). It is important to note that HuNoVs that exhibit identical, related, or closely binding patterns tend to cluster together (31). Thus, it has been shown that HBGA binding patterns play an important role in the evolution of HuNoV.

## The Replicative Cycle of HuNoV

The HuNoV replicative cycle starts with the interaction of the virus with the target cell through the recognition of specific cellular receptors and, in some cases, other cellular components. In the case of HuNoV, the main receptor is still unknown. However, some co-receptors and cell-binding factors have been described, including HBGA and bile acids among others (87). HuNoV binds to the cell by the interaction of the P2 region present in the *P* domain of the VP1 protein with a still unknown receptor and some host co-receptors such as HBGA (55).

After the interaction of VP1 with the cell receptor(s), internalization of the virus and subsequent disassembly of the viral capsid occur, releasing the RNA into the cell cytoplasm. The exact mechanism by which these two processes take place has not yet been described. Once the viral genome is released, the VPg protein, which is covalently linked at the 5′ end of the viral genome, interacts with the cellular translation initiation factors, such as eIF3, generating a translation complex (67). This activity has been described in several caliciviruses, suggesting that this function is highly conserved (67, 89). Subsequently, the major and the minor ribosomal subunits are recruited, resulting in the translation of the non-structural polyprotein which contains the non-structural proteins of the virus (67, 68). Once the non-structural polyprotein of HuNoV is generated, the protease is auto-cleaved. Subsequently, the protease co- and post-translationally cleaves the rest of the polyprotein, generating three protein precursors: p48/NTPase, p22/VPg, and Pro/Pol (31, 37, 61). Currently, the enzymatic functions of the p48/NTPase and p22/VPg complexes have not been described. However, the ProPol precursor has two enzymatic functions: protease and polymerase activities. It should be noted that the ProPol complex has a higher enzymatic performance compared to the activity of both proteins separately (75, 77, 90). Subsequently, the three precursors are cleaved by the action of the ProPol complex, which in turn self-cleaves and generates the six individual non-structural proteins. These individual proteins have specific functions in the replicative and the infective cycles.

The replication process is carried out by the action of Pol, VPg, and NTPase proteins. The latter has three activities: helicase, NTP hydrolase, and chaperone (32, 62). Subsequently, the p48 protein will be integrated into this process, which will enhance the Pol activity (57). Then, synthesis of the viral genome and the sub-genomes with the VPg protein, attached at their 5′ ends, which recruits the cellular translation initiation factors will occur (67). Finally, both the genome and the sub-genomes will be mobilized by the NTPase protein chaperone activity (62, 89). A viral polyprotein will be produced from the translation of the viral genome, and VP1 and VP2 proteins will be generated from the sub-genomes (33, 82). Once the three proteins have been generated, the assembly and the release of the virions take place through a mechanism that remains to be elucidated (33, 91).

**Figure 2:**
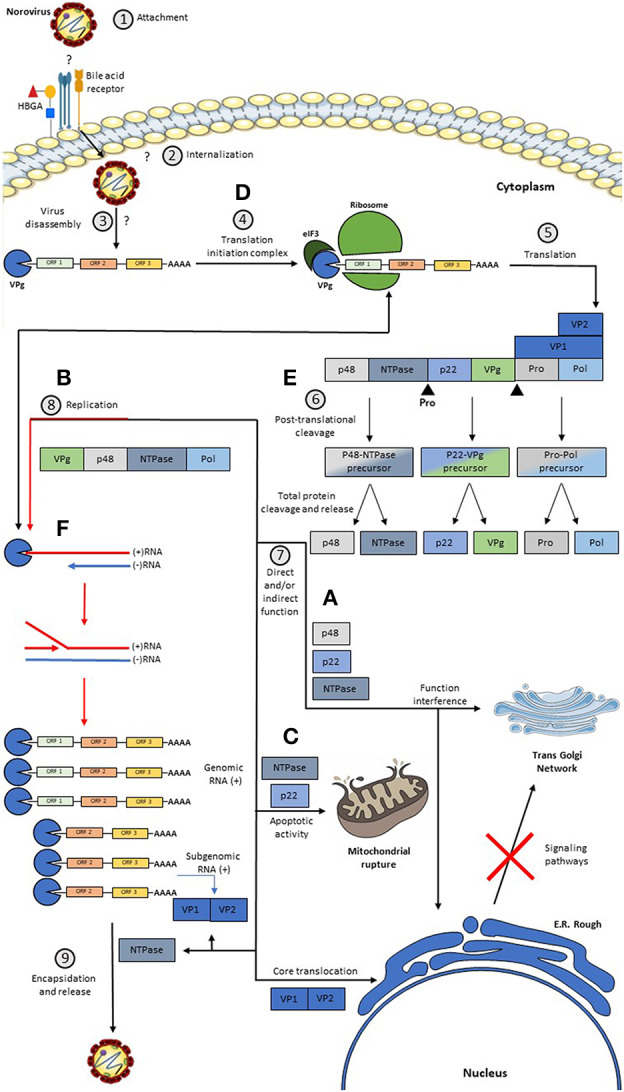
Model of a Replicative HuNoV Cycle and Function of its Proteins. (1) The replication process begins with the binding of the P2 region present in the *P* domain of VP1 to a still unknown receptor and some host co-receptors such as HBGA; (2, 3) After this union there is an internalization of the virus in the cell and a disassembly of the virus releasing the RNA in the cell cytoplasm; (4, 5) once in the cytoplasm, the covalently linked VPg protein at the 5′ end induces the binding of translation initiation factors, such as eIF3, and the binding of major and minor ribosomal subunits, producing genome translation viral thus generating a non-structural polyprotein, the VP1 protein, and the VP2 protein; (6) after this, the Pro protein cleaves the polyprotein generating three precursors, but only the Pro-Pol precursor has enzymatic activity, these precursors are subsequently cleaved generating the six individual proteins; (7) subsequently: **(A)** The P48 protein migrates to the reticulum and subsequently to the Golgi. **(B)** The NTPase protein will help in the replication process. **(C)** The P22 protein binds to the vesicles coated with COPII and contributes synergistically with the disassembly of the Golgi, and this protein together with the NTPase acts favoring the pro-apoptotic activity of the cell. **(D)** the VPg protein participates both in the recruitment of translation initiation factors, and with the help of the replication process, in addition to this, it has a replication complex formation function. **(E)** Pro protein has a non-structural polyprotein cleavage activity. **(F)** The Pol protein exerts its function in viral replication, whose activity is greatly enhanced by the action of P48. On the other hand, the VP1 and VP2 proteins are generated through a subgenomic RNA, which after translation takes place the assembly of the virions and subsequently, the output.

While this process occurs, the p48 protein migrates to the endoplasmic reticulum and to the trans-Golgi network where its disassembly is induced (30), causing interference of the signaling pathways of NFκB, MAPK, and PI3K-Akt, which are important for the host's immune response (29, 30). p48 binds to protein A that is associated with VAP-A, which participates in SNARE-mediated vesicular transport and causes a blockage in cell protein transport (30, 35). Besides that, the effect caused by p48 is reinforced by the action of p22, which synergistically contributes to the disassembly of the trans-Golgi network (36). P22 also blocks the traffic of COPII-coated vesicles since it has a motif that closely resembles an export signal from the ER (36). p22 and NTPase favor the pro-apoptotic activity of the cell, facilitating the release of HuNoV virions from the host cell (82, 91, 92). A proposed model of the HuNoV replicative cycle in human enterocytes is described in Figure 2.

## Immune Response to HuNoV

The immunological response to HuNoV in humans is complex, considering the large variability of circulating genotypes and the pre-existing exposures to these viruses (93). Short-term immunity has been evidenced against HuNoV. Nevertheless, the exact molecular mechanism behind these responses remains to be elucidated.

### The Innate Immune Response Against HuNoV

Two main cell pathways of the innate immune system are raised against RNA viruses such as HuNoV, the type I and III interferon (IFN) systems controlling viral replication (94, 95). Particularly, RNA viruses are mainly detected by the pattern recognition receptors (PRRs), which include the TLR and the RIG-I like receptor (RLR) family members. TLR3 is localized in the intracellular compartment in macrophages, B lymphocytes, and cDCs and it has been characterized as one of the principal sensors of RNA viruses (95). RIG-I and MDA5, which belong to the RLR family, are likewise cytoplasmic sensors that detect intracellular RNA viruses. Current understanding indicates that TLR3 recognizes any dsRNA in the endosome compartments, while MDA5 recognizes long dsRNA and RIG-I senses triphosphate containing dsRNA in the cytoplasm (95).

Only a few reports suggest that the effectors of the IFN pathways limit HuNoV replication. Specifically, IFN-α has been reported to play a role in controlling HuNoV replication in Huh-7 cells and BHK21 cells, suggesting that IFN production may be one of the host antiviral mechanisms in controlling HuNoV infection (96). Furthermore, the following evidences suggest that the type I and III IFN systems can play an important role in controlling HuNoV infections: (a) high IFN-α cytokine production has been detected in the intestinal contents and the serum of pigs inoculated with a GII.4 strain (97); (b) the replication of MNV-1 is sensitive to the type I and III IFN systems both *in vivo* and *in vitro* (98, 99); (c) NV replication in Huh-7 cells, after transfection with genomic NV RNA, is inhibited when cells are pre-treated with the supernatant from cells that have been transfected with the IFN inductor, poly inosinic/polycytidylic acid (poly I:C), to induce IFNα/β production (100); and (d) bile acids allow the replication of GI.1, GII.3, and GII.17 HuNoV strains and enhance it in GII.4 variants in human intestinal enteroids (20). Interestingly, bile acids have previously been reported to down-regulate type I and III IFN pathways and enhance the growth of the porcine enteric calicivirus (101). The latter strongly suggests that the downregulation of these IFN pathways allows and enhances HuNoV infection in human cells, although it has been challenging to prove it. Additionally, permissive NV genome replication after transfecting NV RNA in Huh-7 cells does not induce strong type I IFN responses (102). Using the NV RNA transfection method described by Guix et al. it was likewise recently reported that HuNoV does not induce type I or type III IFN pathway in the human cell line 293FT (103). Nevertheless, further studies are required since these cell types are not necessarily suitable to evaluate these IFN responses.

Moreover, in a recent study by Dang et al. (104) using a HuNoV (NV) replicon system, it was possible to evaluate the antiviral activities of different types of IFNs. Specifically, this study showed that HuNoV replication was sensitive to the three types of IFNs: type I IFN (IFNα), type II IFN (IFNγ), and type III IFN (IFNλ1 and 3). Furthermore, by expressing different interferon-stimulated genes (ISGs) into the human Huh7 hepatocellular carcinoma cells expressing the NV replicon (HG23), RTP4, and HPSE were identified, which showed moderate anti-NV effector activities. Additionally, it was found that the transcription factor IRF-1, with RIG-I, and MDA-5 were induced in the HGC3 cells treated with types I and III IFNs. Likewise, IRF-1 was predominantly induced in HGC3 by treating them with type II IFN. Interestingly, these investigators demonstrated that there is a synergic effect of the different IFN systems. Specifically, it was observed that these ISGs were induced coordinately by the three IFN systems in effectively controlling HuNoV replication. It was also found that NV did not impede the antiviral response induced by these different types of IFNs (104). This is the first report to show the key molecules involved in the three IFN systems to inhibit HuNoV replication. Further studies are required for understanding HuNoV–host interactions so as to design proper antiviral therapies against these viruses.

### The Adaptive Immune Response to HuNoV

The adaptive immune response against HuNoV is barely understood. However, some studies have provided insights into how the humoral and the cellular immune responses are elicited to these viruses in humans.

#### Humoral Immune Response

Rapidly evolving RNA viruses, such as GII.4 HuNoV, elicit complex humoral responses associated with previous exposure.

In general, it is not clearly defined whether serum antibody levels against HuNoV are correlated with protection against future infections by these viruses. Several studies in the literature indicate that high levels of serum antibodies against NV in adults correlate with protection against future infections by this virus (GI.1 genotype) (105, 106). Likewise, the results of other studies, conducted in adult volunteers, revealed that the presence of high serum or fecal titers of specific antibodies for NV before infection decreased the probability of becoming infected by this virus, compared to the case of volunteers with low titers of pre-existing antibodies (105, 107, 108). Additionally, in children, high serum antibody levels appear to be correlated with protection, possibly due to a short-term immunity and recent exposures to these viruses (109, 110). However, in a challenge study carried out with early re-exposure to NV, 50% of the volunteers infected with this HuNoV had repeated infections after 3 years compared to the other 50% who remained without infection during and after repeated challenges (111). The problem of this study was that it could not be demonstrated whether these volunteers were exempt from disease due to acquired immunity or their genetic resistance traits to the virus. Nevertheless, these evidences suggest that frequent exposure to HuNoV can stimulate resistance to HuNoV-induced diseases (105, 106). One possible mechanism behind these observations is that multiple exposures to HuNoV may also induce IgA antibody production, which is known to play a role in short-term immune protection against viruses (112). In support of this notion, it has also been shown that mucosal IgA is produced in the small intestine tissues of NV-infected volunteers (113). Further studies are required to fully elucidate the mechanism behind the short-term immunity to HuNoV.

On the other hand, no evidence of long-term protective immunity has been proven for these viruses. Indeed some volunteers who became ill after a NV challenge showed partial immunity against the disease in a re-exposure after 6–14 weeks. However, this partial immune protection was completely lost after 2–3 years (111). Nevertheless, long-term protective immune response can be evidenced from numerous epidemiological reports from different parts of the world that show periods of “high norovirus activity,” which correlate with the apparition of new GII.4 strains (114–118). This phenomenon is followed by a period of years with reduced numbers of HuNoV outbreaks, which suggests a generation of herd immunity in the population that protects against GII.4 HuNoV infections (46, 119, 120). However, it is important to keep in mind that individuals can become susceptible again to the disease if a new strain of GII.4 appears.

Furthermore, the correlation between neutralizing antibody titers and protection against future infections is also unclear. It has been shown that the serum antibodies in volunteers are capable of blocking the binding of NV VLPs to HBGAs (121, 122). Specifically, it was observed that only 20–30% of people who had pre-existing anti-NV antibodies had pre-existing blocking antibody titers. Moreover, between 90 and 100% of those volunteers, after the challenge with NV, generated blocking antibody titers (121, 122). However, none of these studies has tested a correlation between blocking antibody titers and clinical outcomes. Interestingly, the convalescent cross-blocking serum of the GI VLP–HBGA interaction was tested and it was found that each volunteer demonstrated a unique pattern of VLP blocking (122). Similarly, other studies have shown a high activity of blocking the binding of GII.4 VLPs to HBGA H type 3 in the serum of uninfected children during a waterborne outbreak of acute gastroenteritis, suggesting that strong blocking activity correlated with protection against HuNoV (123, 124). Moreover, an early study showed that strains from different genogroups are antigenically dissimilar (112). Specifically, it was demonstrated that a NV (GI.1) failed to confer immunity to subsequent disease caused by the Hawaii virus (GII.1) and the opposite. In contrast, NV conferred immunity to a later challenge with the Montgomery County virus (GI.5), suggesting an antigenic relatedness between genotypes within a genogroup (112). However, it is not always the case since by measuring cross-reactivity and cross-blockade activity, using serum samples from HuNoV-infected patients, limited cross-reactivity was found but no cross-blockade activity between GII.4 and GII.17, suggesting that GII.4-specific immunity does not provide efficient protection against the GII.17 genotype (125).

In summary, humoral protection after HuNoV infection has very complex patterns of heterotypic immune responses. To date, no study has demonstrated that the presence of high cross-reactivity and cross-blockade activities in the serum of HuNoV-infected individuals guarantees long-term protection against future infections by these viruses, most likely due to the rapid molecular evolution of the HuNoV capsid gene.

#### Cellular Immune Response

Most of the studies that have been conducted in trying to understand the immune response raised to HuNoV have focused on the antibody-mediated response (84, 126). Besides that, no suitable small animal model is available for studying the adaptive immune to HuNoV. Therefore, limited information is available regarding the cellular immune response against these viruses.

Nevertheless, there is evidence of a specific cellular immune response raised to HuNoV. Indeed in the serum samples of healthy volunteers infected with G1.1, increased levels of IFN-γ, as well as interleukin (IL)-10 and IL-12p70 among other cytokines produced during a cellular immune response, have been found (127).

Moreover, significant increases of IFN-γ (Th1 cytokine) have also been reported in serum samples from volunteers challenged with the Snow Mountain Virus (SMV) and the GII reference strain (GII.2-1976) or in fecal samples from travelers with HuNoV gastroenteritis (128). Particularly, peripheral blood mononuclear cells (PBMCs) from healthy volunteers challenged with the SMV had a significant rise of IFN-γ production after *in vitro* stimulation with the same HuNoV VLP (128). A smaller, but significant, increase above the pre-challenge titer was also detected for IL-2 (Th1 cytokine) and IL-5 (Th2 cytokine). In contrast, significant changes between pre-challenge and post-challenge PBMC secretion of TNF-α, IL-10, or IL-4 were not detected, suggesting a predominant, but not exclusive, Th1 cellular immune response to GII HuNoV challenge (128). Also, these investigators aimed to identify the cellular source of the Th1 cytokines produced in response to the SMV challenge and found that CD4^+^ cells were the primary source of IFN-γ secretion in PBMCs stimulated *in vitro* with SMV VLPs. Similarly, the cellular immune response to GI HuNoV was also studied (122). Specifically, PBMCs from six out of 10 NV (GI.1-1968 strain)-infected volunteers, after *in vitro* VLP stimulation with different homologous or heterologous GI strains (GI.1-1968, GI.1-2001, GI.2-1999, GI.3-1999, and GI.4-2000), had a significant increase of IFN-γ secretion to at least one GI VLP stimulation. Interestingly, the cellular immune response was found to be preferentially targeting an alternative GI VLP rather than the infecting strain (122). These data suggest that a Th1 response is mounted against homologous and heterologous GI HuNoV strains.

In summary, the immune response induced by HuNoVs, which are rapidly evolving RNA viruses, has been difficult to achieve. Indeed the ability of these viruses to mutate, in key residues of immunodominant epitopes over time, suggests an evolution of the viral capsid, allowing HuNoVs not only to escape from the immune response of the host's adaptive immune response but also to adapt their binding to any of the HBGAs. A full understanding of the humoral and the cellular immune responses to HuNoV still remains to be elucidated.

## Conclusions

The study of the function of HuNoV proteins has been hindered because, for decades, there was no efficient cell culture system for these viruses. However, various investigators have carried out studies of the HuNoV structural proteins. VP1, which can self-assemble and form VLPs, has facilitated the development of candidate vaccines based on these structures. In contrast, the development of research based on NS proteins of HuNoV has been limited to *in vitro* cultures that have provided information about their role in the replication and the pathogenesis of HuNoV.

The structural protein VP1 is one of the most relevant HuNoV proteins because the P domain, specifically the P2 subdomain, is very variable and has a higher selective pressure, constantly inducing mutations to avoid the host's immune response. Due to these variations, this protein is used for the classification of HuNoV genogroups and genotypes. Also, in the P2 subdomain, there are sites for cell-binding receptors and co-receptors, such as the HBGA and bile acids. In contrast, protein VP2 acts as a stabilizer of the viral capsid and, during viral replication, it enhances the expression of VP1 protein and modulates the activity of RdRp along with VP1 and p48 proteins.

Host susceptibility factors, such as HBGAs, are essential for HuNoV infection. The *fut2* gene determines the secretory and the non-secretory characteristics of those individuals who present or do not present the active form of the FUT2 enzyme, respectively. The specific patterns by which HuNoV binds to these HBGAs can be classified into two groups: (i) those with HBGA type A/B binding patterns that recognize A and/or B and H antigens, but not the antigens of Lewis and (ii) those that have Lewis antigen binding patterns and bind only with the Lewis and/or H antigens.

Although the HuNoV viral replicative cycle has not been fully clarified due to the limitation in knowledge about the functions of the HuNoV proteins, we propose their roles in the HuNoV replicative model as follows: (1) VPg plays a role in recruiting cellular translation initiation factors; (2) NS proteins are released from the generated polyprotein by the viral protease, which is self-cleaved first; (3) p48 and p22 function in interfering cell signaling pathways; (4) VP1 and VP2 function synergistically, increasing their transduction rates; (5) RdRp, VP1, NTPase, and p48 work coordinately in the HuNoV replication process; (6) NTPase acts as a chaperone protein; and (7) NTPase and p48 proteins may have pro-apoptotic activities.

Additionally, the study of the immune response against HuNoV has been challenging due to the great variety of circulating genotypes and the constant evolution of these strains. The high rate of mutation of VP1 is what allows the alteration in the antigenicity regions of the virus. Although there is no clear evidence on the development of long-term protection against HuNoV, the generation of short-term protection has been evidenced by different studies, resulting in a high frequency of reinfection cases. A Th1 response and the activation of IFN-γ production have also been described upon HuNoV infection, which is suggested to play a role in the immune response to this pathogen.

A better understanding of HuNoV pathogenesis and the immune response raised to these viruses will make possible a suitable design and the development of therapeutic aids, including vaccines for controlling HuNoV infections.

## Author Contributions

CC-V, JC, AA, and ML contributed to the conception and design of the manuscript. CC-V, JC, and AA performed bibliographic search and writing of the manuscript. ML, AK, and CC critically reviewed the manuscript. DE wrote the first draft of the manuscript. JC and CC contributed to the manuscript figures. All authors contributed to the revision of the manuscript, read, and approved the version presented.

## Conflict of Interest

The authors declare that the research was conducted in the absence of any commercial or financial relationships that could be construed as a potential conflict of interest.

## References

[1] Fankhauser RL Monroe SS Noel JS Humphrey CD Bresee JS Parashar UD. Epidemiologic and molecular trends of “Norwalk-like viruses” associated with outbreaks of gastroenteritis in the United States. J Infect Dis. (2002) 186:1–7. 10.1086/341085 12089655

[2] Glass RI Parashar UD Estes MK. Norovirus gastroenteritis. N Engl J Med. (2009) 361:1776–85. 10.1056/NEJMra0804575 19864676 PMC3880795

[3] Bartsch SM Lopman BA Ozawa S Hall AJ Lee BY. Global economic burden of norovirus gastroenteritis. PLoS ONE. (2016) 11:e0151219. 10.1371/journal.pone.0151219 27115736 PMC4846012

[4] Hall AJ Lopman BA Payne DC Patel MM Gastanaduy PA Vinje J Norovirus disease in the United States. Emerg Infect Dis. (2013) 19:1198–205. 10.3201/eid1908.130465 23876403 PMC3739528

[5] Mead PS Slutsker L Dietz V McCaig LF Bresee JS Shapiro C. Food-related illness and death in the United States. Emerg Infect Dis. (1999) 5:607–25. 10.3201/eid0505.990502 10511517 PMC2627714

[6] Lopman BA Reacher M Gallimore C Adak GK Gray JJ Brown DW. A summertime peak of “winter vomiting disease”: surveillance of noroviruses in England and Wales, 1995 to 2002. BMC Public Health. (2003) 3:13. 10.1186/1471-2458-3-13 12659651 PMC153520

[7] Fankhauser RL Noel JS Monroe SS Ando T Glass RI. Molecular epidemiology of “Norwalk-like viruses” in outbreaks of gastroenteritis in the United States. J Infect Dis. (1998) 178:1571–8. 10.1086/314525 9815206

[8] Roddie C Paul JP Benjamin R Gallimore CI Xerry J Gray JJ. Allogeneic hematopoietic stem cell transplantation and norovirus gastroenteritis: a previously unrecognized cause of morbidity. Clin Infect Dis. (2009) 49:1061–8. 10.1086/605557 19705974

[9] Westhoff TH Vergoulidou M Loddenkemper C Schwartz S Hofmann J Schneider T. Chronic norovirus infection in renal transplant recipients. Nephrol Dial Transplant. (2009) 24:1051–3. 10.1093/ndt/gfn693 19073655

[10] Ajami N Koo H Darkoh C Atmar RL Okhuysen PC Jiang ZD. Characterization of norovirus-associated traveler's diarrhea. Clin Infect Dis. (2010) 51:123–30. 10.1086/653530 20540620 PMC3150512

[11] Koo HL Ajami NJ Jiang ZD Neill FH Atmar RL Ericsson CD. Noroviruses as a cause of diarrhea in travelers to Guatemala, India, and Mexico. J Clin Microbiol. (2010) 48:1673–6. 10.1128/JCM.02072-09 20305012 PMC2863947

[12] Graham DY Jiang X Tanaka T Opekun AR Madore HP Estes MK. Norwalk virus infection of volunteers: new insights based on improved assays. J Infect Dis. (1994) 170:34–43. 10.1093/infdis/170.1.34 8014518

[13] Chhabra P de Graaf M Parra GI Chan MC Green K Martella V. Updated classification of norovirus genogroups and genotypes. J Gen Virol. (2019) 100:1393–406. 10.1099/jgv.0.001318 31483239 PMC7011714

[14] Cannon JL Lindesmith LC Donaldson EF Saxe L Baric RS Vinje J. Herd immunity to GII.4 noroviruses is supported by outbreak patient sera. J Virol. (2009) 83:5363–74. 10.1128/JVI.02518-08 19297483 PMC2681945

[15] Vinje J Altena SA Koopmans MP. The incidence and genetic variability of small round-structured viruses in outbreaks of gastroenteritis in The Netherlands. J Infect Dis. (1997) 176:1374–8. 10.1086/517325 9359742

[16] Tamminen K Malm M Vesikari T Blazevic V. Immunological cross-reactivity of an ancestral and the most recent pandemic norovirus GII.4 variant. Viruses. (2019) 11:91. 10.3390/v11020091 30678195 PMC6410201

[17] Lindesmith LC Brewer-Jensen PD Mallory ML Debbink K Swann EW Vinjé J. Antigenic characterization of a novel recombinant GII. P16-GII. 4 Sydney norovirus strain with minor sequence variation leading to antibody escape. J Infect Dis. (2017) 217:1145–52. 10.1093/infdis/jix651 29281104 PMC5939617

[18] Lindesmith LC Kocher JF Donaldson EF Debbink K Mallory ML Swann EW. Emergence of novel human norovirus GII.17 strains correlates with changes in blockade antibody epitopes. J Infect Dis. (2017) 216:1227–34. 10.1093/infdis/jix385 28973354 PMC5853573

[19] Jones MK Grau KR Costantini V Kolawole AO de Graaf M Freiden P. Human norovirus culture in B cells. Nat Protoc. (2015) 10:1939–47. 10.1038/nprot.2015.121 26513671 PMC4689599

[20] Ettayebi K Crawford SE Murakami K Broughman JR Karandikar U Tenge VR. Replication of human noroviruses in stem cell-derived human enteroids. Science. (2016) 353:1387–93. 10.1126/science.aaf5211 27562956 PMC5305121

[21] Van Dycke J Ny A Conceicao-Neto N Maes J Hosmillo M Cuvry A. A robust human norovirus replication model in zebrafish larvae. PLoS Pathog. (2019) 15:e1008009. 10.1371/journal.ppat.1008009 31536612 PMC6752765

[22] Cortes-Penfield NW Ramani S Estes MK Atmar RL. Prospects and challenges in the development of a norovirus vaccine. Clin Ther. (2017) 39:1537–49. 10.1016/j.clinthera.2017.07.002 28756066 PMC5776706

[23] Alvarado G Ettayebi K Atmar RL Bombardi RG Kose N Estes MK. Human monoclonal antibodies that neutralize pandemic GII.4 noroviruses. Gastroenterology. (2018) 155:1898–907. 10.1053/j.gastro.2018.08.039 30170116 PMC6402321

[24] Alhatlani B Vashist S Goodfellow I. Functions of the 5' and 3' ends of calicivirus genomes. Virus Res. (2015) 206:134–43. 10.1016/j.virusres.2015.02.002 25678268 PMC4509552

[25] May J Viswanathan P Ng KK-S Medvedev A Korba B. The P4-P2′ amino acids surrounding human norovirus polyprotein cleavage sites define the core sequence regulating self-processing order. J Virol. (2014) 88:10738–47. 10.1128/JVI.01357-14 24991013 PMC4178882

[26] Glass PJ White LJ Ball JM Leparc-Goffart I Hardy ME Estes MK. Norwalk virus open reading frame 3 encodes a minor structural protein. J Virol. (2000) 74:6581–91. 10.1128/JVI.74.14.6581-6591.2000 10864672 PMC112168

[27] Jiang X Wang M Wang K Estes MK. Sequence and genomic organization of Norwalk virus. Virology. (1993) 195:51–61. 10.1006/viro.1993.1345 8391187

[28] Vongpunsawad S Prasad BV Estes MK. Norwalk virus minor capsid protein VP2 associates within the VP1 shell domain. J Virol. (2013) 87:4818–25. 10.1128/JVI.03508-12 23408637 PMC3624303

[29] Lateef Z Gimenez G Baker ES Ward VK. Transcriptomic analysis of human norovirus NS1-2 protein highlights a multifunctional role in murine monocytes. BMC Genomics. (2017) 18:39. 10.1186/s12864-016-3417-4 28056773 PMC5217272

[30] Ettayebi K Hardy ME. Norwalk virus non-structural protein p48 forms a complex with the SNARE regulator VAP-A and prevents cell surface expression of vesicular stomatitis virus G protein. J Virol. (2003) 77:11790–7. 10.1128/JVI.77.21.11790-11797.2003 14557663 PMC229264

[31] Huang P Farkas T Zhong W Tan M Thornton S Morrow AL. Norovirus and histo-blood group antigens: demonstration of a wide spectrum of strain specificities and classification of two major binding groups among multiple binding patterns. J Virol. (2005) 79:6714–22. 10.1128/JVI.79.11.6714-6722.2005 15890909 PMC1112114

[32] Pfister T Wimmer E. Polypeptide p41 of a Norwalk-like virus is a nucleic acid-independent nucleoside triphosphatase. J Virol. (2001) 75:1611–9. 10.1128/JVI.75.4.1611-1619.2001 11160659 PMC114070

[33] Bull RA Hansman GS Clancy LE Tanaka MM Rawlinson WD White PA. Norovirus recombination in ORF1/ORF2 overlap. Emerging Infect Dis. (2005) 11:1079. 10.3201/eid1107.041273 16022784 PMC3371806

[34] Thorne LG Goodfellow IG. Norovirus gene expression and replication. J Gen Virol. (2014) 95:278–91. 10.1099/vir.0.059634-0 24243731

[35] Roth AN Karst SM. Norovirus mechanisms of immune antagonism. Curr Opin Virol. (2016) 16:24–30. 10.1016/j.coviro.2015.11.005 26673810 PMC4821668

[36] Sharp TM Guix S Katayama K Crawford SE Estes MK. Inhibition of cellular protein secretion by norwalk virus non-structural protein p22 requires a mimic of an endoplasmic reticulum export signal. PLoS ONE. (2010) 5:e13130. 10.1371/journal.pone.0013130 20976190 PMC2956632

[37] Belliot G Sosnovtsev SV Chang KO Babu V Uche U Arnold JJ. Norovirus proteinase-polymerase and polymerase are both active forms of RNA-dependent RNA polymerase. J Virol. (2005) 79:2393–403. 10.1128/JVI.79.4.2393-2403.2005 15681440 PMC546540

[38] Mallory ML Lindesmith LC Graham RL Baric RS. GII. 4 human norovirus: surveying the antigenic landscape. Viruses. (2019) 11:177. 10.3390/v11020177 30791623 PMC6410000

[39] Jiang X Wang M Graham DY Estes MK. Expression, self-assembly, and antigenicity of the Norwalk virus capsid protein. J Virol. (1992) 66:6527–32. 10.1128/JVI.66.11.6527-6532.1992 1328679 PMC240146

[40] Prasad BV Rothnagel R Jiang X Estes MK. Three-dimensional structure of baculovirus-expressed Norwalk virus capsids. J Virol. (1994) 68:5117–25. 10.1128/JVI.68.8.5117-5125.1994 8035511 PMC236455

[41] Prasad BV Hardy ME Dokland T Bella J Rossmann MG Estes MK. X-ray crystallographic structure of the Norwalk virus capsid. Science. (1999) 286:287–90. 10.1126/science.286.5438.287 10514371

[42] Bertolotti-Ciarlet A White LJ Chen R Prasad BV Estes MK. Structural requirements for the assembly of Norwalk virus-like particles. J Virol. (2002) 76:4044–55. 10.1128/JVI.76.8.4044-4055.2002 11907243 PMC136079

[43] Tan M Hegde RS Jiang X The P domain of norovirus capsid protein forms dimer and binds to histo-blood group antigen receptors. J Virol. (2004) 78:6233–42. 10.1128/JVI.78.12.6233-6242.2004 15163716 PMC416535

[44] Allen DJ Gray JJ Gallimore CI Xerry J Iturriza-Gomara M. Analysis of amino acid variation in the P2 domain of the GII-4 norovirus VP1 protein reveals putative variant-specific epitopes. PLoS ONE. (2008) 3:e1485. 10.1371/journal.pone.0001485 18213393 PMC2194622

[45] Lindesmith LC Debbink K Swanstrom J Vinje J Costantini V Baric RS. Monoclonal antibody-based antigenic mapping of norovirus GII.4-2002. J Virol. (2012) 86:873–83. 10.1128/JVI.06200-11 22090098 PMC3255811

[46] Lindesmith LC Donaldson EF Lobue AD Cannon JL Zheng DP Vinje J. Mechanisms of GII.4 norovirus persistence in human populations. PLoS Med. (2008) 5:e31. 10.1371/journal.pmed.0050031 18271619 PMC2235898

[47] White LJ Ball JM Hardy ME Tanaka TN Kitamoto N Estes MK. Attachment and entry of recombinant Norwalk virus capsids to cultured human and animal cell lines. J Virol. (1996) 70:6589–97. 10.1128/JVI.70.10.6589-6597.1996 8794293 PMC190699

[48] Zheng DP Ando T Fankhauser RL Beard RS Glass RI Monroe SS. Norovirus classification and proposed strain nomenclature. Virology. (2006) 346:312–23. 10.1016/j.virol.2005.11.015 16343580

[49] Shanker S Choi JM Sankaran B Atmar RL Estes MK Prasad BV. Structural analysis of histo-blood group antigen binding specificity in a norovirus GII.4 epidemic variant: implications for epochal evolution. J Virol. (2011) 85:8635–45. 10.1128/JVI.00848-11 21715503 PMC3165782

[50] Donaldson EF Lindesmith LC Lobue AD Baric RS. Viral shape-shifting: norovirus evasion of the human immune system. Nat Rev Microbiol. (2010) 8:231–41. 10.1038/nrmicro2296 20125087 PMC7097584

[51] Troeger H Loddenkemper C Schneider T Schreier E Epple HJ Zeitz M. Structural and functional changes of the duodenum in human norovirus infection. Gut. (2009) 58:1070–7. 10.1136/gut.2008.160150 19036950

[52] Bull RA Tanaka MM White PA. Norovirus recombination. J Gen Virol. (2007) 88:3347–59. 10.1099/vir.0.83321-0 18024905

[53] Eden JS Tanaka MM Boni MF Rawlinson WD White PA. Recombination within the pandemic norovirus GII.4 lineage. J Virol. (2013) 87:6270–82. 10.1128/JVI.03464-12 23536665 PMC3648122

[54] Cotten M Petrova V Phan MV Rabaa MA Watson SJ Ong SH. Deep sequencing of norovirus genomes defines evolutionary patterns in an urban tropical setting. J Virol. (2014) 88:11056–69. 10.1128/JVI.01333-14 25056894 PMC4178781

[55] Lin Y Fengling L Lianzhu W Yuxiu Z Yanhua J. Function of VP2 protein in the stability of the secondary structure of virus-like particles of genogroup II norovirus at different pH levels: function of VP2 protein in the stability of NoV VLPs. J Microbiol. (2014) 52:970–5. 10.1007/s12275-014-4323-6 25277406

[56] Liu Z Zhang M Shen Z Chen H Zhang W Xu X. The coordinating role of the human norovirus minor capsid protein VP2 is essential to functional change and nuclear localization of the major capsid protein VP1. Arch Virol. (2019) 164:1173–80. 10.1007/s00705-019-04192-2 30810804

[57] Subba-Reddy CV Goodfellow I Kao CC. VPg-primed RNA synthesis of norovirus RNA-dependent RNA polymerases by using a novel cell-based assay. J Virol. (2011) 85:13027–37. 10.1128/JVI.06191-11 21994457 PMC3233154

[58] Fernandez-Vega V Sosnovtsev SV Belliot G King AD Mitra T Gorbalenya A. Norwalk virus N-terminal nonstructural protein is associated with disassembly of the Golgi complex in transfected cells. J Virol. (2004) 78:4827–37. 10.1128/JVI.78.9.4827-4837.2004 15078964 PMC387691

[59] Hardy ME Crone TJ Brower JE Ettayebi K. Substrate specificity of the Norwalk virus 3C-like proteinase. Virus Res. (2002) 89:29–39. 10.1016/S0168-1702(02)00114-4 12367748

[60] Liu B Clarke IN Lambden PR. Polyprotein processing in Southampton virus: identification of 3C-like protease cleavage sites by *in vitro* mutagenesis. J Virol. (1996) 70:2605–10. 10.1128/JVI.70.4.2605-2610.1996 8642693 PMC190109

[61] Belliot G Sosnovtsev SV Mitra T Hammer C Garfield M Green KY *In vitro* proteolytic processing of the MD145 norovirus ORF1 non-structural polyprotein yields stable precursors and products similar to those detected in calicivirus-infected cells. J Virol. (2003) 77:10957–74. 10.1128/JVI.77.20.10957-10974.2003 14512545 PMC224964

[62] Li TF Hosmillo M Schwanke H Shu T Wang Z Yin L. Human norovirus NS3 has RNA helicase and chaperoning activities. J Virol. (2018) 92:17. 10.1128/JVI.01606-17 29237842 PMC5809735

[63] Yen JB Wei LH Chen LW Chen LY Hung CH Wang SS. Subcellular localization and functional characterization of GII.4 norovirus-encoded NTPase. J Virol. (2018) 92:17. 10.1128/JVI.01824-17 29212938 PMC5809722

[64] Doerflinger SY Cortese M Romero-Brey I Menne Z Tubiana T Schenk C. Membrane alterations induced by non-structural proteins of human norovirus. PLoS Pathog. (2017) 13:e1006705. 10.1371/journal.ppat.1006705 29077760 PMC5678787

[65] Hyde JL Mackenzie JM. Subcellular localization of the MNV-1 ORF1 proteins and their potential roles in the formation of the MNV-1 replication complex. Virology. (2010) 406:138–48. 10.1016/j.virol.2010.06.047 20674956

[66] Belliot G Sosnovtsev SV Chang KO McPhie P Green KY. Nucleotidylylation of the VPg protein of a human norovirus by its proteinase-polymerase precursor protein. Virology. (2008) 374:33–49. 10.1016/j.virol.2007.12.028 18234264 PMC2386983

[67] Daughenbaugh KF Fraser CS Hershey JW Hardy ME. The genome-linked protein VPg of the Norwalk virus binds eIF3, suggesting its role in translation initiation complex recruitment. EMBO J. (2003) 22:2852–9. 10.1093/emboj/cdg251 12773399 PMC156748

[68] Hosmillo M Lu J McAllaster MR Eaglesham JB Wang X Emmott E. Noroviruses subvert the core stress granule component G3BP1 to promote viral VPg-dependent translation. Elife. (2019) 8:e46681. 10.7554/eLife.46681 31403400 PMC6739877

[69] McSweeney A Davies C Ward VK. Cell cycle arrest is a conserved function of norovirus VPg proteins. Viruses. (2019) 11:217. 10.3390/v11030217 30836641 PMC6466040

[70] Nakamura K Someya Y Kumasaka T Ueno G Yamamoto M Sato T. A norovirus protease structure provides insights into active and substrate binding site integrity. J Virol. (2005) 79:13685–93. 10.1128/JVI.79.21.13685-13693.2005 16227288 PMC1262588

[71] Blakeney SJ Cahill A Reilly PA. Processing of Norwalk virus non-structural proteins by a 3C-like cysteine proteinase. Virology. (2003) 308:216–24. 10.1016/S0042-6822(03)00004-7 12706072

[72] Chang KO Kim Y Lovell S Rathnayake AD Groutas WC. Antiviral drug discovery: norovirus proteases and development of inhibitors. Viruses. (2019) 11:197. 10.3390/v11020197 30823509 PMC6410195

[73] Zeitler CE Estes MK Venkataram Prasad BV. X-ray crystallographic structure of the Norwalk virus protease at 1.5-A resolution. J Virol. (2006) 80:5050–8. 10.1128/JVI.80.10.5050-5058.2006 16641296 PMC1472067

[74] Someya Y Takeda N Miyamura T. Characterization of the norovirus 3C-like protease. Virus Res. (2005) 110:91–7. 10.1016/j.virusres.2005.02.002 15845259 PMC7114197

[75] Viskovska MA Zhao B Shanker S Choi J-M Deng L Song Y. GII. 4 norovirus protease shows pH-sensitive proteolysis with a unique Arg-His pairing in the catalytic site. J Virol. (2019) 93:e01479–18. 10.1128/JVI.01479-18 30626675 PMC6401421

[76] Scheffler U Rudolph W Gebhardt J Rohayem J. Differential cleavage of the norovirus polyprotein precursor by two active forms of the viral protease. J Gen Virol. (2007) 88:2013–8. 10.1099/vir.0.82797-0 17554035

[77] May J Korba B Medvedev A Viswanathan P. Enzyme kinetics of the human norovirus protease control virus polyprotein processing order. Virology. (2013) 444:218–24. 10.1016/j.virol.2013.06.013 23850457

[78] Ng KK-S Pendás-Franco N Rojo J Boga JA Machín À Alonso JMM. Crystal structure of Norwalk virus polymerase reveals the carboxyl terminus in the active site cleft. J Biol Chem. (2004) 279:16638–45. 10.1074/jbc.M400584200 14764591

[79] Ozaki K Matsushima Y Nagasawa K Motoya T Ryo A Kuroda M. Molecular evolutionary analyses of the RNA-dependent RNA polymerase region in norovirus genogroup II. Front Microbiol. (2018) 9:3070. 10.3389/fmicb.2018.03070 30619155 PMC6305289

[80] Mallagaray A Creutznacher R Dulfer J Mayer PHO Grimm LL Orduna JM. A post-translational modification of human Norovirus capsid protein attenuates glycan binding. Nat Commun. (2019) 10:1320. 10.1038/s41467-019-09251-5 30899001 PMC6428809

[81] de Graaf M van Beek J Koopmans MP. Human norovirus transmission and evolution in a changing world. Nat Rev Microbiol. (2016) 14:421–33. 10.1038/nrmicro.2016.48 27211790

[82] Cao S Lou Z Tan M Chen Y Liu Y Zhang Z. Structural basis for the recognition of blood group trisaccharides by norovirus. J Virol. (2007) 81:5949–57. 10.1128/JVI.00219-07 17392366 PMC1900264

[83] Hansman GS Shahzad-Ul-Hussan S McLellan JS Chuang GY Georgiev I Shimoike T. Structural basis for norovirus inhibition and fucose mimicry by citrate. J Virol. (2012) 86:284–92. 10.1128/JVI.05909-11 22031945 PMC3255897

[84] Lindesmith L Moe C Marionneau S Ruvoen N Jiang X Lindblad L. Human susceptibility and resistance to Norwalk virus infection. Nat Med. (2003) 9:548–53. 10.1038/nm860 12692541

[85] Hemming M Räsänen S Huhti L Paloniemi M Salminen M Vesikari T. Major reduction of rotavirus, but not norovirus, gastroenteritis in children seen in hospital after the introduction of RotaTeq vaccine into the National Immunization Programme in Finland. Eur J Pediatr. (2013) 172:739–46. 10.1007/s00431-013-1945-3 23361964 PMC7086648

[86] Ramani S Giri S. Influence of histo blood group antigen expression on susceptibility to enteric viruses and vaccines. Curr Opin Infect Dis. (2019) 32:445–52. 10.1097/QCO.0000000000000571 31335438

[87] Marionneau S Ruvoen N Le Moullac-Vaidye B Clement M Cailleau-Thomas A Ruiz-Palacois G. Norwalk virus binds to histo-blood group antigens present on gastroduodenal epithelial cells of secretor individuals. Gastroenterology. (2002) 122:1967–77. 10.1053/gast.2002.33661 12055602 PMC7172544

[88] Huang P Farkas T Marionneau S Zhong W Ruvoën-Clouet N Morrow AL. Noroviruses bind to human ABO, Lewis, and secretor histo-blood group antigens: identification of 4 distinct strain-specific patterns. J Infect Dis. (2003) 188:19–31. 10.1086/375742 12825167

[89] Shirato H Ogawa S Ito H Sato T Kameyama A Narimatsu H. Noroviruses distinguish between type 1 and type 2 histo-blood group antigens for binding. J Virol. (2008) 82:10756–67. 10.1128/JVI.00802-08 18701592 PMC2573190

[90] Medvedev A Viswanathan P May J Korba B. Regulation of human norovirus VPg nucleotidylylation by ProPol and nucleoside triphosphate binding by its amino terminal sequence *in vitro*. Virology. (2017) 503:37–45. 10.1016/j.virol.2017.01.003 28110248

[91] Hutson AM Atmar RL Graham DY Estes MK. Norwalk virus infection and disease is associated with ABO histo-blood group type. J Infect Dis. (2002) 185:1335–7. 10.1086/339883 12001052

[92] Thorne LG Goodfellow IG. Norovirus gene expression and replication. J Gen Virol. (2014) 95:278–91. 10.1099/vir.0.059634-0 24243731

[93] Donaldson EF Lindesmith LC Lobue AD Baric RS. Norovirus pathogenesis: mechanisms of persistence and immune evasion in human populations. Immunol Rev. (2008) 225:190–211. 10.1111/j.1600-065X.2008.00680.x 18837783

[94] Lee S Baldridge MT. Interferon-lambda: a potent regulator of intestinal viral infections. Front Immunol. (2017) 8:749. 10.3389/fimmu.2017.00749 28713375 PMC5491552

[95] Jensen S Thomsen AR. Sensing of RNA viruses: a review of innate immune receptors involved in recognizing RNA virus invasion. J Virol. (2012) 86:2900–10. 10.1128/JVI.05738-11 22258243 PMC3302314

[96] Chang KO Sosnovtsev SV Belliot G King AD Green KY. Stable expression of a Norwalk virus RNA replicon in a human hepatoma cell line. Virology. (2006) 353:463–73. 10.1016/j.virol.2006.06.006 16843517

[97] Souza M Cheetham SM Azevedo MS Costantini V Saif LJ. Cytokine and antibody responses in gnotobiotic pigs after infection with human norovirus genogroup II.4 (HS66 strain). J Virol. (2007) 81:9183–92. 10.1128/JVI.00558-07 17581999 PMC1951422

[98] Karst SM Wobus CE Lay M Davidson J Virgin HWt. STAT1-dependent innate immunity to a Norwalk-like virus. Science. (2003) 299:1575–8. 10.1126/science.1077905 12624267

[99] Wobus CE Karst SM Thackray LB Chang KO Sosnovtsev SV Belliot G. Replication of Norovirus in cell culture reveals a tropism for dendritic cells and macrophages. PLoS Biol. (2004) 2:e432. 10.1371/journal.pbio.0020432 15562321 PMC532393

[100] Guix S Asanaka M Katayama K Crawford SE Neill FH Atmar RL. Norwalk virus RNA is infectious in mammalian cells. J Virol. (2007) 81:12238–48. 10.1128/JVI.01489-07 17855551 PMC2168986

[101] Chang KO Sosnovtsev SV Belliot G Kim Y Saif LJ Green KY. Bile acids are essential for porcine enteric calicivirus replication in association with down-regulation of signal transducer and activator of transcription 1. Proc Natl Acad Sci USA. (2004) 101:8733–8. 10.1073/pnas.0401126101 15161971 PMC423264

[102] Brasier AR García-Sastre A Lemon SM Cellular signaling and innate immune responses to RNA virus infections. Washington, DC: ASM Press (2009). 10.1128/9781555815561

[103] Qu L Murakami K Broughman JR Lay MK Guix S Tenge VR. Replication of human norovirus RNA in mammalian cells reveals lack of interferon response. J Virol. (2016) 90:8906–23. 10.1128/JVI.01425-16 27466422 PMC5021416

[104] Dang W Xu L Yin Y Chen S Wang W Hakim MS. IRF-1, RIG-I and MDA5 display potent antiviral activities against norovirus coordinately induced by different types of interferons. Antiviral Res. (2018) 155:48–59. 10.1016/j.antiviral.2018.05.004 29753657

[105] Johnson PC Mathewson JJ DuPont HL Greenberg HB. Multiple-challenge study of host susceptibility to Norwalk gastroenteritis in US adults. J Infect Dis. (1990) 161:18–21. 10.1093/infdis/161.1.18 2153184

[106] Okhuysen PC Jiang X Ye L Johnson PC Estes MK. Viral shedding and fecal IgA response after Norwalk virus infection. J Infect Dis. (1995) 171:566–9. 10.1093/infdis/171.3.566 7876602

[107] Gray J Cunliffe C Ball J Graham DY Desselberger U Estes MK. Detection of immunoglobulin M (IgM), IgA, and IgG Norwalk virus-specific antibodies by indirect enzyme-linked immunosorbent assay with baculovirus-expressed Norwalk virus capsid antigen in adult volunteers challenged with Norwalk virus. J Clin Microbiol. (1994) 32:3059–63. 7883902 10.1128/jcm.32.12.3059-3063.1994 PMC264229

[108] Baron RC Greenberg HB Cukor G Blacklow NR. Serological responses among teenagers after natural exposure to Norwalk virus. J Infect Dis. (1984) 150:531–4. 10.1093/infdis/150.4.531 6092484

[109] Lew JF Valdesuso J Vesikari T Kapikian AZ Jiang X Estes MK Detection of Norwalk virus or Norwalk-like virus infections in Finnish infants and young children. J Infect Dis. (1994) 169:1364–7. 10.1093/infdis/169.6.1364 8195618

[110] Chachu KA LoBue AD Strong DW Baric RS Virgin HW. Immune mechanisms responsible for vaccination against and clearance of mucosal and lymphatic norovirus infection. PLoS Pathogens. (2008) 4:236. 10.1371/journal.ppat.1000236 19079577 PMC2587711

[111] Parrino TA Schreiber DS Trier JS Kapikian AZ Blacklow NR. Clinical immunity in acute gastroenteritis caused by Norwalk agent. N Engl J Med. (1977) 297:86–9. 10.1056/NEJM197707142970204 405590

[112] Wyatt RG Dolin R Blacklow NR DuPont HL Buscho RF Thornhill TS. Comparison of three agents of acute infectious non-bacterial gastroenteritis by cross-challenge in volunteers. J Infect Dis. (1974) 129:709–14. 10.1093/infdis/129.6.709 4209723

[113] Agus SG Dolin R Wyatt RG Tousimis AJ Northrup RS Acute infectious non-bacterial gastroenteritis: intestinal histopathology. Histologic and enzymatic alterations during illness produced by the Norwalk agent in man. Ann Intern Med. (1973) 79:18–25. 10.7326/0003-4819-79-1-18 4721173

[114] Adamson WE Gunson RN Maclean A Carman WF. Emergence of a new norovirus variant in Scotland in 2006. J Clin Microbiol. (2007) 45:4058–60. 10.1128/JCM.01853-07 17942666 PMC2168558

[115] Johansen K Mannerqvist K Allard A Andersson Y Burman LG Dillner L. Norovirus strains belonging to the GII. 4 genotype dominate as a cause of nosocomial outbreaks of viral gastroenteritis in Sweden 1997–2005: Arrival of new variants is associated with large nation-wide epidemics. J Clin Virol. (2008) 42:129–34. 10.1016/j.jcv.2007.12.012 18304864

[116] Okada M Ogawa T Yoshizumi H Kubonoya H Shinozaki K. Genetic variation of the norovirus GII-4 genotype associated with a large number of outbreaks in Chiba prefecture, Japan. Arch Virol. (2007) 152:2249–52. 10.1007/s00705-007-1028-8 17851731

[117] Reuter G Pankovics P Szucs G. Genetic drift of norovirus genotype GII-4 in seven consecutive epidemic seasons in Hungary. J Clin Virol. (2008) 42:135–40. 10.1016/j.jcv.2008.02.014 18420454

[118] Tu ET-V Bull RA Greening GE Hewitt J Lyon MJ Marshall JA. Epidemics of gastroenteritis during 2006 were associated with the spread of norovirus GII. 4 variants 2006a and 2006b. Clin Infect Dis. (2008) 46:413–20. 10.1086/525259 18177226

[119] Siebenga JJ Vennema H Duizer E Koopmans MP. Gastroenteritis caused by norovirus GGII. 4, The Netherlands, 1994–2005. Emerging Infect Dis. (2007) 13:144. 10.3201/eid1301.060800 17370531 PMC2913659

[120] Siebenga JJ Vennema H Renckens B de Bruin E van der Veer B Siezen RJ. Epochal evolution of GGII.4 norovirus capsid proteins from 1995 to 2006. J Virol. (2007) 81:9932–41. 10.1128/JVI.00674-07 17609280 PMC2045401

[121] Reeck A Kavanagh O Estes MK Opekun AR Gilger MA Graham DY. Serological correlate of protection against norovirus-induced gastroenteritis. J Infect Dis. (2010) 202:1212–8. 10.1086/656364 20815703 PMC2945238

[122] Lindesmith LC Donaldson E Leon J Moe CL Frelinger JA Johnston RE. Heterotypic humoral and cellular immune responses following Norwalk virus infection. J Virol. (2010) 84:1800–15. 10.1128/JVI.02179-09 20007270 PMC2812379

[123] Rockx BH Vennema H Hoebe CJ Duizer E Koopmans MP. Association of histo-blood group antigens and susceptibility to norovirus infections. J Infect Dis. (2005) 191:749–54. 10.1086/427779 15688291

[124] Nurminen K Blazevic V Huhti L Rasanen S Koho T Hytonen VP. Prevalence of norovirus GII-4 antibodies in Finnish children. J Med Virol. (2011) 83:525–31. 10.1002/jmv.21990 21264875

[125] Dai Y-C Xia M Huang Q Tan M Qin L Zhuang Y-L. Characterization of antigenic relatedness between GII. 4 and GII. 17 noroviruses by use of serum samples from norovirus-infected patients. J Clin Microbiol. (2017) 55:3366–73. 10.1128/JCM.00865-17 28904188 PMC5703803

[126] Hutson AM Airaud F LePendu J Estes MK Atmar RL. Norwalk virus infection associates with secretor status genotyped from sera. J Med Virol. (2005) 77:116–20. 10.1002/jmv.20423 16032732

[127] Newman KL Moe CL Kirby AE Flanders WD Parkos CA Leon JS. Human norovirus infection and the acute serum cytokine response. Clin Exp Immunol. (2015) 182:195–203. 10.1111/cei.12681 26178578 PMC4608509

[128] Lindesmith L Moe C Lependu J Frelinger JA Treanor J Baric RS. Cellular and humoral immunity following Snow Mountain virus challenge. J Virol. (2005) 79:2900–9. 10.1128/JVI.79.5.2900-2909.2005 15709009 PMC548455

